# ACE2 overexpression in corticotropin-releasing-hormone cells offers protection against pulmonary hypertension

**DOI:** 10.3389/fnins.2023.1223733

**Published:** 2023-08-10

**Authors:** Aline C. Oliveira, Marianthi M. Karas, Matthew Alves, Jacky He, Annette D. de Kloet, Eric G. Krause, Elaine M. Richards, Andrew J. Bryant, Mohan K. Raizada

**Affiliations:** ^1^Division of Pulmonary, Critical Care and Sleep Medicine, College of Medicine, University of Florida, Gainesville, FL, United States; ^2^Evelyn F. and William L. McKnight Brain Institute, University of Florida, Gainesville, FL, United States; ^3^Department of Pharmacodynamics, College of Pharmacy, University of Florida, Gainesville, FL, United States; ^4^Department of Physiology and Aging, College of Medicine, University of Florida, Gainesville, FL, United States; ^5^Center for Integrative Cardiovascular and Metabolic Diseases, University of Florida, Gainesville, FL, United States

**Keywords:** pulmonary hypertension, dysautonomia, chronic hypoxia, brain-lung axis, neuroinflammation, neuroimmune interaction

## Abstract

**Background:**

Pulmonary hypertension (PH), characterized by elevated pulmonary pressure and right heart failure, is a systemic disease involving inappropriate sympathetic activation and an impaired gut-brain-lung axis. Global overexpression of angiotensin converting enzyme 2 (ACE2), a cardiopulmonary protective enzyme of the renin-angiotensin system, attenuates PH induced by chronic hypoxia. Neurons within the paraventricular nucleus of the hypothalamus (PVN) that synthesize corticotropin-releasing hormone (CRH) are activated by stressors, like hypoxia, and this activation augments sympathetic outflow to cardiovascular tissues. These data coupled with our observations that ACE2 overexpression in CRH cells (CRH-ACE2KI mice) decreases anxiety-like behavior via suppression of hypothalamic–pituitary–adrenal (HPA) axis activity by decreasing CRH synthesis, led us to hypothesize that selective ACE2 overexpression in CRH neurons would protect against hypoxia-induced PH.

**Methods:**

CRH-ACE2KI and WT male and female mice were exposed to chronic hypoxia (10%O2) or normoxia (21%O2) for 4 weeks in a ventilated chamber with continuous monitoring of oxygen and carbon dioxide concentrations (*n* = 7–10/group). Pulmonary hemodynamics were measured with Millar pressure catheters then tissues were collected for histological analyses.

**Results:**

Chronic hypoxia induced a significant increase (36.4%) in right ventricular (RV) systolic pressure (RVSP) in WT mice, which was not observed in CRH-ACE2KI mice. No significant differences in RVSP were observed between male and female mice in any of the groups.

**Conclusion:**

Overexpression of ACE2 in CRH cells was protective against hypoxia-induced PH. Since the majority of expression of CRH is in brain nuclei such as paraventricular nucleus of the hypothalamus (PVN) and/or central nucleus of the amygdala (CeA) these data indicate that the protective effects of ACE2 are, at least in part, centrally mediated. This contributes to the systemic nature of PH disease and that CRH neurons may play an important role in PH.

## 1. Introduction

Pulmonary hypertension (PH) is a progressive disease that leads to right heart failure and death (Lettieri et al., 2006; Collard et al., 2012). Dysautonomia contributing to PH progression is associated with a worse prognosis due to a series of well-established downstream sequelae, including ventricular dysfunction, remodeling, arrhythmia, and pulmonary arterial vasoconstriction(Meredith et al., 1991; Francis et al., 1993; Velez-Roa et al., 2004; Ishikawa et al., 2009; Wensel et al., 2009; Ciarka et al., 2010; Vaillancourt et al., 2017). However, the mechanism behind the chronic elevation in sympathetic activity in PH is poorly understood (Klinger, 2016).

The renin-angiotensin-system (RAS) has been implicated in the pathophysiology of PH. While increased angiotensin (Ang) II was correlated with mortality in patients with idiopathic PH (de Man et al., 2012), the beneficial effect of the RAS counterregulatory axis, represented by angiotensin converting enzyme 2 (ACE2) and Ang-(1–7) have been explored in clinical trials (ClinicalTrials.gov identifier: NCT01884051 and NCT03304548). So far, targeting ACE2 in animal models have shown significant improvement in PH outcomes, such as attenuation of pulmonary pressure, cardiac hypertrophy, pulmonary vascular remodeling, neuroinflammation and dysautonomia (Ferreira et al., 2009, 2011; Sharma et al., 2020; Oliveira et al., 2022).

Prevailing evidence, accumulated over the last several decades, supports the involvement of autonomic imbalance in the progression of PH. Increased sympathetic activity leads to cardiac hypertrophy, arrhythmias, vasoconstriction, remodeling, and apoptosis, that aggravate PH (Meredith et al., 1991; Velez-Roa et al., 2004; Wensel et al., 2009; Ciarka et al., 2010; Vaillancourt et al., 2017). Clinical trials implementing pulmonary arterial denervation (PADN) have found improved ECG and hemodynamic profiles in PH patients (Vaillancourt et al., 2017; Constantine and Dimopoulos, 2021; Leopold, 2022; Zhang et al., 2022a,b); however, such an intervention is not without risks and the mechanisms underlying a sustained increase in sympathetic activation remain unclear. Initial studies into the origin of dysautonomia in PH proposed a chemoreflex response to dyspnea, a common symptom of PH. Surprisingly, Velez-Roa et al. (2004) found that peripheral chemoreflex deactivation by hyperoxia (100% oxygen) led to a decrease of 25% of sympathetic activity in PH patients. This data suggests that the main component contributing to chronically increased sympathetic activity in PH patients involves a more nuanced mechanism, potentially including crosstalk within the brain nuclei governing autonomic outflow.

Corticotropin-releasing hormone (CRH) neurons, well-known mediators of the HPA axis, are key in the autonomic response to stress (Wang et al., 2018), receive dense input from the nucleus of the solitary tract (NTS) and are the most highly activated neurons in the PVN following acute hypoxia exposure (Ruyle et al., 2018). Additionally, Dikme (2018) proposed serum cortisol as a putative marker to predict major adverse pulmonary events in adults with acute nontraumatic dyspnea. Furthermore, there is a robust association of variations in the CRH receptor 1 (CRHR1) and CRH-binding protein (CRHBP) in neonates with hypoxemic respiratory failure and clinical diagnosis of persistent PH of the newborn (PPHN). However, the extent and mechanisms of CRH involvement in the development of PH remains elusive.

We have previously reported that ACE2 overexpression restricted to CRH synthesizing cells leads to suppression of CRH synthesis, blunting of HPA axis activation and attenuation of anxiety-like behavior. Since we have been evaluating the mechanisms involved in autonomic disbalance in PH and the contribution of a brain-lung axis to this pathophysiology, we hypothesized that ACE2 overexpression on CRH cells would be sufficient to promote protection against PH.

## 2. Methods

### 2.1. Animals

All experimental procedures were approved by the University of Florida Institute Animal Care and Use Committee. A total of 41 mice were used for the study; 8 male C57CL6J exposed to normoxia (*n* = 4) or chronic hypoxia (*n* = 4); 17 CRH ACE2 KI (8 male and 9 female) and 16 littermate controls (7 male and 9 female). Mice used for the study were housed in a temperature-controlled room (22°C to 25°C) with a 12:12-h light dark cycle. They had access to autoclaved standard mouse chow, sterile water *ad libitum* and housed in sterile corn cob bedding. Male and female mice ranged in weight from 20 to 30 g and were 8–12 weeks old at the initiation of the study.

In order to test the hypothesis that ACE2 overexpression in CRH cells leads to protection against PH induced by chronic hypoxia, we used CRH ACE2KI mice that were previously described in Wang et al. (2018). In brief, mice homozygous for the floxed STOP ACE2 gene (i.e., the ACE2 gene was preceded by a floxed STOP codon at the ROSA26 locus) and those expressing CRH-Cre knock-in modification (Jackson Laboratory Stock # 012704) were crossed, such that litters contained CRH ACE2KI mice expressing both genetic modifications and littermate controls that carry only the floxed STOP ACE2 gene (WT mice).

Mice were exposed to either room air (FiO2 21%) or chronic hypoxia (FiO2 10%) inside a normobaric chamber (Coy Laboratory Products) with continuous monitoring of oxygen and carbon dioxide concentrations. Carbon dioxide was removed by ventilation and activated charcoal filtration through an air purifier, which maintained carbon dioxide at less than 0.1%. Exposure to normal air was limited to water, food and cage changes twice a week. After 28 days, mice were anesthetized for hemodynamic measurements and histological examination.

### 2.2. Hemodynamic measurements

First, animals were anesthetized with 125 mg/kg of Avertin (tert-amyl alcohol and 2,2,2-tribromoethanol; Thermo ScientificTM #AC421432500) in PBS. Throughout the recording of less than 10 min duration, animals breathed room air, without necessity of artificial ventilation. Direct right ventricular systolic pressure (RVSP) measurement was performed in a terminal procedure using a 1.4-French-pressure-volume catheter transducer (Millar Instruments, SPR-671) connected to a signal processor (PowerLab/8 s and ADInstruments). The catheter was inserted through a right internal jugular vein incision and threaded down into the right ventricle. After 5 min of stable measurements, RVSP (mmHg), a surrogate marker for pulmonary artery pressure and positive and negative right ventricular contractility (dP/dt) were analyzed: subsequently, animals were euthanized for harvest of tissues.

### 2.3. *In situ* hybridization

We performed RNAscope *in situ* hybridization in the PVN, a brain nucleus containing cells that synthesize CRH, using previously validated and described procedures 20. Reagents used for RNAscope *in situ* hybridization were purchased from Advanced Cell Diagnostics (Newark, CA, United States). Briefly, C57BL6J mice exposed to normoxia/chronic hypoxia (*n* = 4/group) were euthanized with an overdose of isoflurane and transcardially perfused with RNase free saline, followed by 4% RNase free paraformaldehyde (PFA). Brains were quickly extracted, submerged in RNase free 4% PFA for 4 h, and then transferred into RNase free 30% sucrose at 4°C. Brains were coronally sectioned at 30 μm. Mounted brain sections were air-dried for 20 min at room temperature. On the day of conducting RNAscope *in situ* hybridization, brain sections were incubated in protease IV (Catalog No. 322340), then hybridized with specific probes following procedures described in the RNAscope® Multiplex Fluorescent Kit User Manual PART 2 (Advanced Cell Diagnostics, Newark, CA, United States). The specific probes used in this study were: (1) DapB, negative control, (2) Ubc, positive control, (3) CRH (Catalog No. 316091). Then sections were co-stained with anti-Iba1 antibody and DAPI to label microglia and nuclei, respectively. Images were examined on a Nikon Eclipse Ni C2+ laser scanning confocal microscope (Nikon Instruments Inc., Melville, NY, United States). Z-Stack confocal images were used to evaluate the expression of CRH mRNA and quantification of Iba1 intensity was evaluated by integrated density using Fiji (ImageJ).

### 2.4. Histological staining and analysis

Following hemodynamic measurements, 17 CRH ACE2 KI and 16 control littermates were perfused transcardially with 20 mL of phosphate buffered saline and 20 mL of 4% neutral buffered paraformaldehyde. Brain, heart and lung tissues were removed for subsequent morphometry and histological analyses.

#### 2.4.1. Lungs

Fixed lungs were paraffin embedded, sectioned with a Leica RM2235 Microtome, and stained for α-smooth muscle actin and Masson Trichrome to assess inflammation and to identify muscularized pulmonary vessels as previously described by Fu et al. (2021). Images were taken with Keyence BZ-X microscope at 10x magnification. Image processing was performed using BZ-X-Analyzer software (Keyence) and quantification performed in Fiji (ImageJ).

α-Smooth muscle actin (αSMA) staining and muscularized vessel counting: Formalin-fixed lung sections were stained for rabbit polyclonal αSMA (Abcam #ab5694; diluted 1:750 in antibody diluent reagent solution [Life Technologies] in blocking reagent Background Sniper [Biocare]). Vessels were considered small if ≤50 μM, medium if 50–150 μM, or large if >150 μM. Median wall thickness of small vessels were quantified at 5 different points/vessel (magnification 10x) from each mouse. Muscularized pulmonary vessels were visualized by brown staining.

Masson Trichrome Staining (MTC) and inflammation scoring: Lung inflammation was evaluated using a 0 to 4 scale as described previously (Lawson et al., 2005): 0, normal lung architecture; 1, increased thickness of some (≤50%) of interalveolar septa; 2, thickening of >50% of interalveolar septa without formation of fibrotic foci; 3, thickening of the interalveolar septa with formation of isolated fibrotic foci; and 4, formation of multiple fibrotic foci with total or subtotal distortion of parenchymal architecture. Evaluation was performed on 10 randomized sequential, non-overlapping fields (magnification 10x) of lung parenchyma from each mouse. Each plotted point represents the mean score for the 10 fields analyzed/mouse.

#### 2.4.2. Brain

Fixed brains were transferred to 30% sucrose for 48 h, sectioned with a Leica cryostat. PVN sections were cut from −0.46 to −1.22 antero-posterior from bregma, then, mounted and stained with DAPI to identify sections containing the PVN. Parallel sections containing the PVN were submitted to Iba1 staining. Free-floating coronal brain sections (40 μm) were pretreated with 1% Triton X-100 in PBS and blocked with 3% normal goat serum. Sections were incubated with primary Iba1 antibody (Wako, #019-19741; rabbit-anti-Iba-1, 1:500) overnight at 4°C, and then stained with goat anti-rabbit Alexa-488 conjugated secondary antibody (Invitrogen # A-11008) for 2 h at room temperature (RT). Sections were rinsed, mounted with Vectashield (Vector Laboratories) and examined on a Nikon Eclipse Ni C2+ laser scanning confocal microscope (Nikon Instruments Inc., Melville, NY, United States). Z-Stack confocal images were used to evaluate the expression of Iba1+ cells and quantification was performed using Fiji (ImageJ).

### 2.5. Statistical analysis

Comparisons among groups were assessed using two-way ANOVA followed by Tukey’s multiple comparison *post hoc* tests. Group data were expressed as mean ± SEM and *p* values <0.05 was considered significant. GraphPad Prism 8.0 (La Jolla, CA, United States) software was used to analyze the data and for graph generation.

## 3. Results

**Figure 1:**
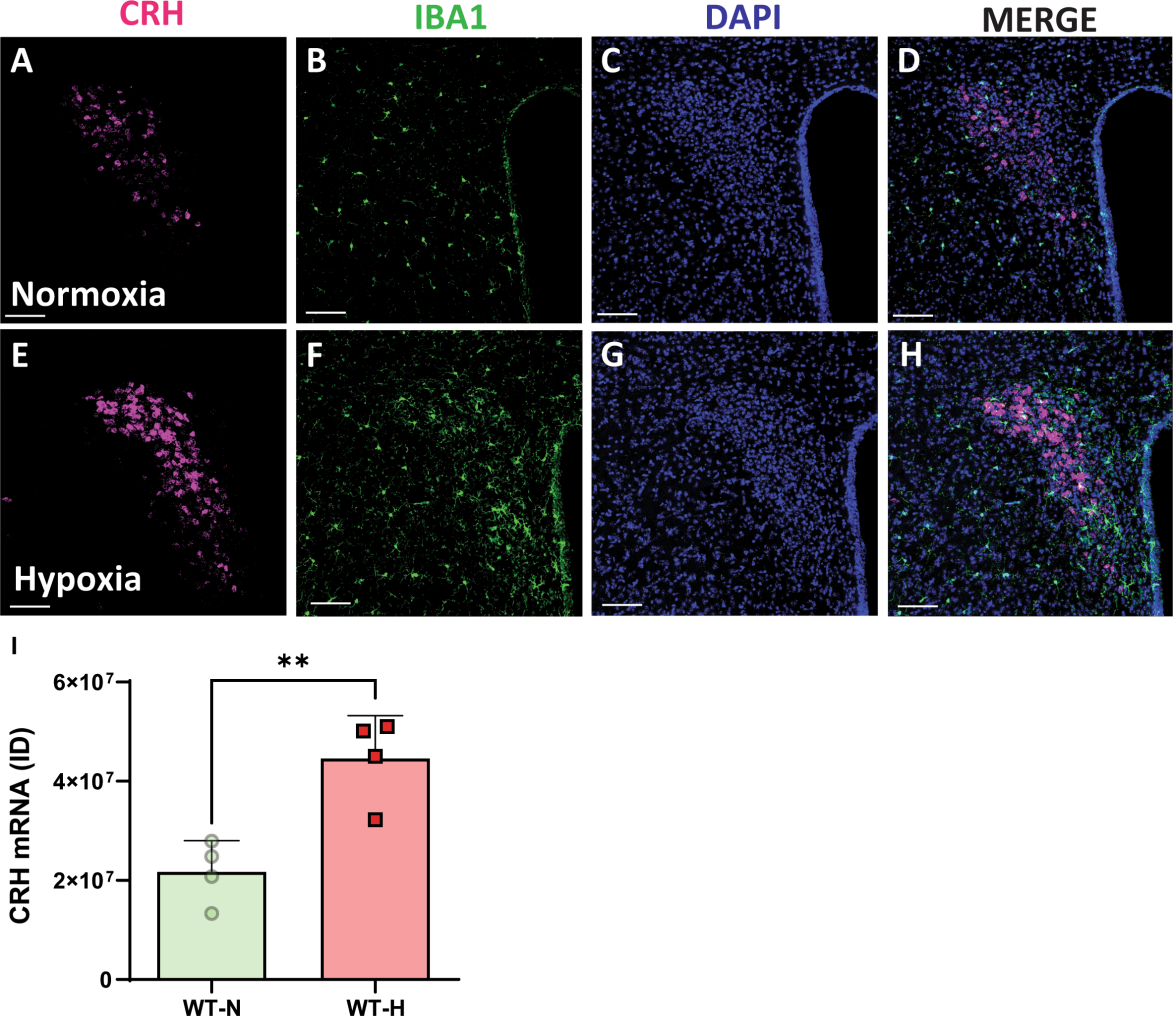
Chronic hypoxia induces a significant increase in mRNA expression for CRH in the PVN. **(A,E)** mRNA expression of CRH (punctate magenta dots) in the paraventricular nucleus of hypothalamus (PVN) of mouse exposed to normoxia (superior panel) and a chronic hypoxia exposed mouse (inferior panel). **(B,F)** Iba1 staining in green. **(C,G)** DAPI staining in blue. **(D,H)** Merged channels. **(I)** Integrated density of ACE2 mRNA signal within the PVN. **p < 0.003. Scale bar: 100 μm.

To investigate how chronic hypoxia impacts CRH expression, we conducted RNAscope *in situ* hybridization for CRH mRNA in the PVN. Control mice had modest levels of CRH mRNA in the PVN (Figures 1A-D) compared with CRH mRNA levels in animals exposed to chronic hypoxia (Figures 1E-H). We previously reported that CRH ACE2 KI mice show significantly increased expression of ACE2 mRNA in CRH-producing regions of the PVN, leading to significant attenuation of CRH synthesis (Wang et al., 2018). Here, we show for the first time that chronic hypoxia causes a significant increase of CRH synthesis in the PVN (Figures 1I) and that ACE2 overexpression in CRH expressing cells leads to protection against chronic hypoxia induced PH.

**Figure 2:**
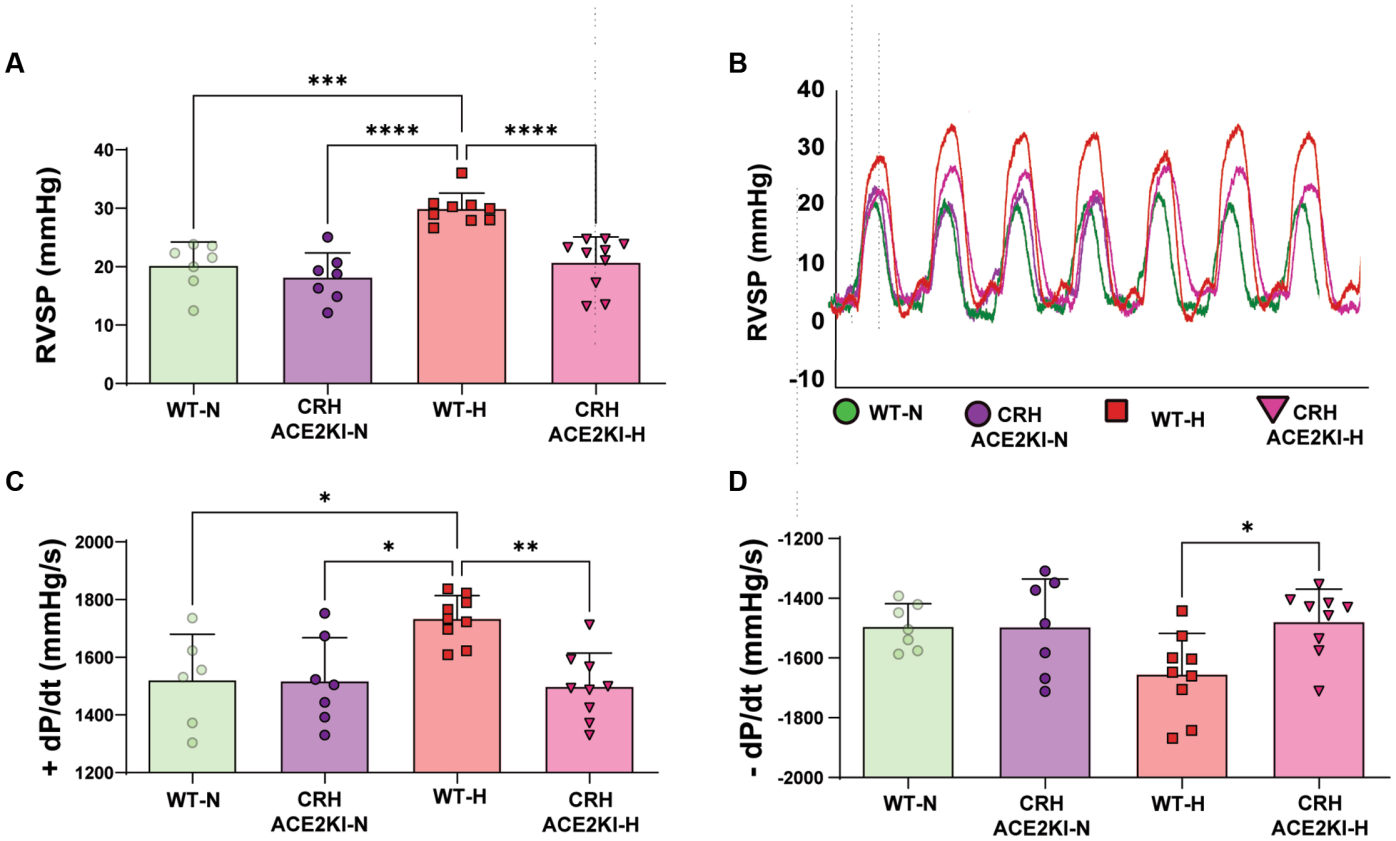
ACE2 (Angiotensin-converting enzyme 2) overexpression on corticotropin-releasing-hormone neurons prevents hypoxia-induced pulmonary hypertension. **(A)** Right ventricular systolic pressure (RVSP) shown in wild-type (WT) and ACE2 knockin (ACE2KI) mice that were either exposed to room air (red) or to chronic hypoxia (purple). **(B)** RVSP representative tracer. **(C)** Right ventricular (RV) contractility as shown by +dP/dt and **(D)** −dP/dt. ****p < 0.001, ***p < 0.01, **p < 0.003, *p < 0.05 (*n* = 7–9 mice/group).

Chronic hypoxia treatment resulted in significant increases in RVSP in WT mice (Normoxia (N):20.18 ± 1.5 mmHg vs. Hypoxia (H):29.89 ± 0.8, *p* < 0.0001). No significant differences in hemodynamic parameters were observed in CRH ACE2KI mice compared to WT in normoxia conditions (RVSP: WT-N:20.18 ± 1.5 mmHg vs. CRH ACE2KI-N: 18.16 ± 1.5 mmHg). Strikingly, mice that overexpress ACE2 exclusively on CRH producing cells were protected against PH (RVSP: WT-H: 29.89 ± 0.8 mmHg vs. CRH ACE2KI-H: 20.68 ± 1.4 mmHg; Figures 2A,B). Similar protection against hypoxia was observed in male and female mice (RVSP: CRH ACE2KI-H: male: 21.5 ± 2.7 mmHg vs. female: 19.35 ± 1.7 mmHg). Similarly, no significant differences were observed between male and female mice in WT mice. Therefore, graphs represent the combined data of female and male mice in all groups.

Associated changes in contractility vs. relaxation capabilities of the RV in WT mice were observed in WT mice exposed to hypoxia, as indicated by changes in + and – dp/dt (+dp/dt: WT-N:1520 ± 65 mmHg/s vs. WT-H: 1733 ± 26 mmHg/s and -dp/dt: WT-N: −1495 ± 28 mmHg/s vs. WT-H: −1655 ± 45 mmHg/s). These results confirm abnormal RV contractility and relaxation, which mimics changes observed in PH patients assessed with similar right heart catheterization (Murch et al., 2015). CRH ACE2KI mice showed attenuated hypoxia-induced changes in right ventricle contractility +dP/dt (*p* < 0.01 versus WT-H, Figure 2C), RV − dP/dt (*p* < 0.01 versus WT-H, Figure 2D), indicating protection of right heart function.

**Figure 3:**
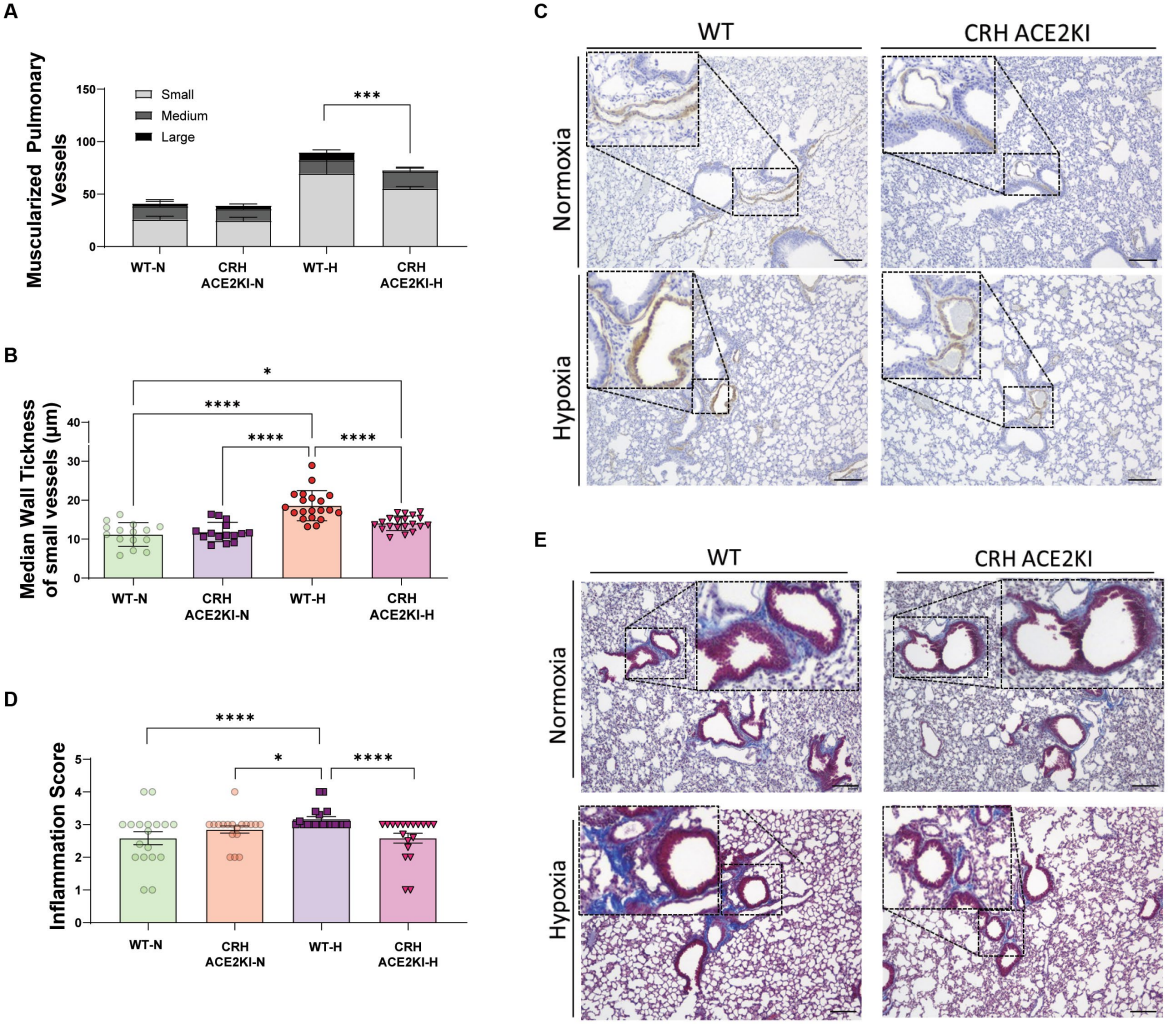
ACE2 overexpression in CRH neurons attenuates smooth muscle cell in pulmonary vessels and lung fibrosis and inflammation induced by chronic hypoxia. **(A,B)** Quantification of muscularized pulmonary vessels (small, medium, large, complete and partial) from IHC staining of α smooth muscle actin (αSMA, brown). Column represents mean ± SEM. Each data point represents the median wall thickness measured/small vessel. Normoxia (*n* = 3/group) and Hypoxia (*n* = 4/group). **(C)** Representative images of αSMA IHC staining on formalin-fixed lung sections. Scale bar: 150 μm at 10x magnification. **(D)** Inflammation and fibrosis scoring represented on Column mean ± SEM of scored data: lung inflammation was evaluated on trichrome-stained lung sections using a 0–4 scale: 0, normal lung architecture; 1, increased thickness of some (≤50%) of interalveolar septa; 2, thickening of >50% of interalveolar septa without formation of fibrotic foci; 3, thickening of the interalveolar septa with formation of isolated fibrotic foci; and 4, formation of multiple fibrotic foci with total or subtotal distortion of parenchymal architecture. ****p < 0.001,***p < 0.01, *p < 0.05 by two-way ANOVA followed by Turkey’s multiple comparisons test. **(E)** Representative images of masson trichrome (MTC)-stained formalin-fixed lung sections. Scale bar: 150 μm at 10x magnification. Each data point represents the score of each image included in the analysis: Normoxia (*n* = 3/group) and Hypoxia (*n* = 4/group) × 10 images/animal.

Analysis of the numbers of muscularized pulmonary vessels and medial wall thickness revealed a significant attenuation of proliferation of cells expressing SMA induced by chronic hypoxia in CRH ACE2KI compared to WT mice (Figure 3). Significant decreases in the number of large pulmonary vessels that were muscularized were observed (Figure 3A). There was a trend for small vessel muscularization to be attenuated in CRH ACE2KI mice but this was not statistically significant (*p* = 0.3182). However, closer analysis of the muscularized small vessels demonstrated that chronic hypoxia significantly increased medial wall thickness in WT mice (WT-H: 18.6 ± 0.8 μm versus WT-N: 11.1 ± 0.7 μm, *p* < 0.0001, Figure 3B) but this increase was not observed in CRH ACE2KI mice (14.8 ± 0.4 μm; *p* < 0.0001 versus WT-H).

We performed Masson Trichrome (MTC) staining using a semi-quantitative score to evaluate the thickness of interalveolar septa with/without formation of fibrotic foci and the parenchymal architecture, as previously described (Pugliese et al., 2015; Fu et al., 2021). A significant increase in the inflammation score was observed in WT mice exposed to hypoxia. Surprisingly, overexpression of ACE2 in CRH neurons prevented the development of lung inflammation and deposition of mature collagen, resulting in no differences between ACE2 CRH mice exposed to hypoxia and mice exposed to normoxia.

**Figure 4:**
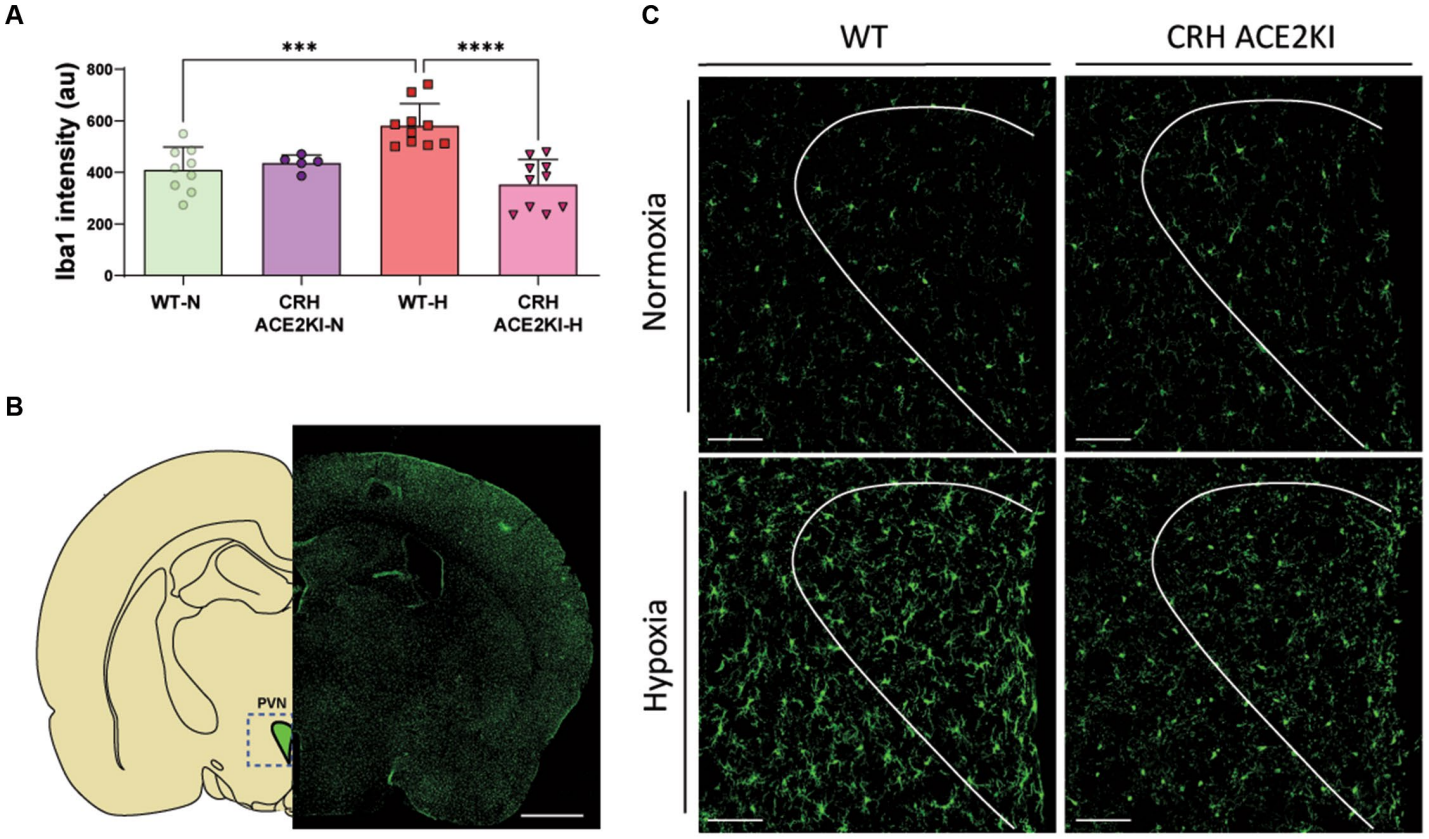
Overexpression of ACE2 on CRH neurons prevented neuroinflammation induced by Chronic Hypoxia. **(A)** Graph bars showing iba1 staining quantification using Fiji (ImageJ). **(B)** Schematic showing PVN region analyzed and Iba1+ staining in green showing microglia expression throughout the brain. Scale bar: 1 mm. **(C)** Confocal micrographs show morphological changes of microglia (Iba1 staining in green) in the PVN following CH-PH in WT mice but not in CRH ACE2KI mice (*n* = 5 mice/group). Scale bar: 100 μm. ****p < 0.001,***p < 0.01 by two-way ANOVA followed by Turkey’s multiple comparisons test.

Finally, we evaluated Iba1positive cells in the PVN since our previous data had shown that hypoxia increases both Iba1 fluorescence and the number of these cells and these were positive correlated with increased RVSP in WT mice. Changes in microglia morphology in WT but not CRH-ACE2KI mice exposed to hypoxia were also compatible with a more pro-inflammatory profile, showing higher Iba1 expression, thicker processes and larger cell bodies. Similarly, only WT mice demonstrated increased Iba1+ cells in the PVN when exposed to chronic hypoxia (Figure 4), indicating that the protection against PH induced by ACE2 overexpression on CRH neurons was concurrent with attenuation of neuroinflammation induced by hypoxia.

## 4. Discussion

Our previous studies established that a global overexpression of ACE2 attenuates chronic hypoxia-induced PH. The significance of the present study is that it demonstrates that ACE2 counter-regulation of CRH-synthesizing cells is sufficient to overcome the hemodynamic changes induced by chronic hypoxia exposure. Pulmonary hypertension is characterized by pulmonary pressures >25 mmHg (King et al., 2013; Ruyle et al., 2019). Our RVSP analysis (Figures 2A,B), serves as a surrogate indicator of pulmonary pressure and reveals that chronic hypoxia exposure induces a significant increase in pulmonary pressure and consequent pulmonary hypertension in WT mice as expected. Strikingly, these changes were completely abolished in CRH ACE2KI mice.

While protection observed in CRH ACE2 KI mice extended to cardiac function by preserving the contractility and relaxation of the right ventricle, the chronic hypoxia model has a moderate effect on cardiac function and does not replicate the complete series of events that leads to RV failure observed in patients with pulmonary hypertension. Since no animal models can fully mimic human PH pathology, future experiments should include other animal models such as Sugen-Hypoxia and monocrotaline-induced PH to validate the contribution of CRH in pathophysiology of PH. Nonetheless, these results indicate that ACE2 overexpression exclusively in CRH-containing cells leads to protection against chronic hypoxia-induced pulmonary hypertension. However, several questions remain concerning the mechanisms involved.

First, shortness of breath is the main debilitating and presenting symptom in pulmonary hypertension and hypoxia leads to several chemoreflex-mediated responses that include increases in ventilation, arterial pressure, neuroendocrine and sympathetic activities, to re-establish and guarantee ideal oxygen supply to tissues. Acute responses to hypoxia involve an extensive integration of brainstem and hypothalamic nuclei, which includes afferent projections from the NTS to the PVN that engage CRH neurons (King et al., 2013; Ruyle et al., 2018, 2019). However, the mechanisms whereby chronic hypoxia exposure leads to a sustained increase in sympathetic activity as observed in pulmonary hypertension, including the neuronal phenotypes involved, are still under investigation. CRH neurons are key mediators of endocrine, behavioral and autonomic responses to stress (Wang et al., 2018). Here, we show, for the first time, that chronic hypoxia exposure leads to significant increase in CRH synthesis in the PVN. Relevant to the current results, intra-PVN release of CRH activates CRHR1(s) expressed on neighboring neurons that send excitatory projections to the rostral ventrolateral medulla (RVLM) (Elsaafien et al., 2021). The RVLM is the primary region of the brainstem that governs sympathetic tone to the vasculature (Turner et al., 2013), leading us to hypothesize that the development of CH-PH may also recruit crosstalk between CRH and RVLM- neurons of the PVN to augment sympathetic outflow. Together with the data presented here, these previous observations reinforce the idea that inhibition of CRH neurons may protect against PH through modulation of the autonomic nervous system.

Furthermore, our data suggest that decreased CRH synthesis through ACE2 overexpression, as observed in CRH ACE2KI mice, can counteract the vascular inflammation induced by CH-PH that can be visualized histologically. ACE2-mediated regulation of CRH synthesis is supported by studies that characterized CRH ACE2KI mice (Wang et al., 2018). Wang and co-authors identified that directing the overexpression of ACE2 to CRH cells attenuates stress-induced HPA axis activation and anxiety-like behavior and decreased the expression of CRH mRNA in the PVN and central nucleus of the amygdala (CeA) (Wang et al., 2018). Interestingly, Zhang and co-authors demonstrated that these CRH expressing neurons in the CeA and PVN are connected to the splenic nerve through a direct neural pathway. While both CRH activation induced by stress or chemogenetics leads to plasma cell formation, ablation or pharmacogenetic inhibition of these neurons reduces plasma cell formation (Zhang et al., 2020). This is particularly important for our understanding of PH pathophysiology since increased inflammation and infiltration of inflammatory cells are established as major contributors for cardiac and pulmonary remodeling in PH. Accordingly, protection observed in CRH ACE2KI mice might be associated with a decreased inflammatory response during the development of CH-PH. Undeniably, these mice showed significant attenuation of lung inflammation, fibrosis and smooth muscle cell proliferation. Indeed, Antalarmin, a CRH receptor antagonist that has been used as a therapeutic tool for CNS and inflammatory disorders has significant anti-inflammatory properties that may be explored as a new pharmacological treatment for pulmonary hypertension.

Microglia are the major contributor to inflammation in the brain. However, microglia have other activities beyond immune functions, displaying interactions with neurons and other glial cells, inducing neurogenesis, synaptic remodeling, pruning and neuronal modulation. Microglia even “remember” prior pro-inflammatory experiences via epigenetic changes, regulating certain forms of respiratory plasticity (Cao et al., 2015; Johnson et al., 2018). Interestingly, Bolton and co-authors demonstrated that early stress exposure induces impaired microglia pruning in CRH-expressing neurons leading to aberrant stress responses in adults (Bolton et al., 2022). Therefore, it is reasonable to consider the possibility that microglia contribute to plasticity in autonomic brain nuclei (i.e., the PVN) that promote sustained increases in sympathetic activity and contribute to PH pathophysiology. Consistent with this notion, a recent report from Qianqian Bi and co-authors have indicated a critical role of microglial cells in the PVN in pre-autonomic neurons (PVN-RVLM) activity (Bi et al., 2022).

Protection observed in CRH ACE2KI mice against hypoxia-induced PH was accompanied by attenuation of Iba1+ cells (marker for microglia/macrophages) in the PVN. We have previously identified neuroinflammation in different models of PH primarily in the PVN. Monocrotaline-induced PH leads to increased pro-inflammatory cytokines such as TNF-α, IL-1β, and IL-6 in the PVN, whereas anti-inflammatory cytokines, such as IL-10, are reduced. The use of two different techniques to promote inhibition of the pro-inflammatory state in microglia by: (1) A pharmacological approach with minocycline, or (2) the exposure of a transgenic mice that lack neuromicroglia interactions through CX3CR1-CX3CL1 showed protection against pulmonary hypertension. Although our current data do not provide a causal/consequence of attenuation of microglia activation, the literature has provided significant evidence of the importance of neuroimmune interactions and its contribution to neuronal modulation. Particularly in PH, we have observed a positive correlation of PH severity and neuroinflammation (microglia activation/increased numbers of Iba1 + cells) (Oliveira et al., 2018). Therefore, the attenuated neuroinflammation observed in CRH ACE2KI mice exposed to hypoxia might be explained by attenuated pathology and therefore attenuated positive feedback to the CNS. However, future studies investigating possible direct mechanisms whereby CRH release may contribute to shifting microglia from homeostatic to pro-inflammatory states are needed.

Another important point to consider is the involvement of the RAS in the pathophysiology of PH and its bidirectional interactions with the autonomic nervous system. The canonical pathway of the RAS is an important physiological regulator of blood pressure and electrolyte balance. Represented by angiotensin converting enzyme (ACE), angiotensin II (Ang II) and angiotensin II type 1 receptor (AT1R), its activation leads to vasoconstriction, pro-inflammatory and pro-fibrotic pathways, contributing significantly to the pathophysiology of several cardiorespiratory diseases, including PH (de Man et al., 2012; Yuan et al., 2015). Conversely, the RAS counter-regulatory limb, ACE2, Ang-(1–7), and Mas receptors, promotes vasodilation, anti-inflammatory and anti-fibrotic pathways that protect against several cardiorespiratory diseases such as PH (Yamazato et al., 2009; Shenoy et al., 2010; Richards and Raizada, 2018; Santos et al., 2018; Oliveira et al., 2022). In the central nervous system, Ang-II/AT1R actions have been implicated in the etiology of anxiety disorders, which may further contribute to chronic activation of the sympathetic nervous system. In contrast, ACE2 overexpression in the CNS reduces anxiety-like behavior through increased frequency of spontaneous inhibitory postsynaptic currents, which indicates presynaptic release of GABA (Wang et al., 2018). Beyond RAS modulation of the autonomic system through neuronal cells within the brain, increasing evidence has supported the RAS canonical pathway as a major contributor to the pro-inflammatory state. Ang II via AT1R increases the release of chemokines and cytokines that influence inflammatory cells and glia. Additionally, Ang II elevates CCL2, which is a proinflammatory chemokine capable of increasing blood brain barrier permeability that is permissive for the infiltration of peripheral circulating immune cells that further induce an incremental pro-inflammatory state.

Taken together, the current data associated with previous reports in the literature led us to propose the existence of a vicious positive feedback cycle in PH, whereby microglial activation contributes to maladaptive neuronal plasticity and chronic sympathetic activation leading to immune cell recruitment to upregulate pro-inflammatory cytokines and contribute to this chronic pro-inflammatory and pro-sympathetic state. This concept is currently under evaluation.

Further investigation of the mechanisms whereby CRH activation may contribute to the pathophysiology of pulmonary hypertension is still needed. Nonetheless, we demonstrated for the first time that attenuated CRH synthesis by overexpression of ACE2 is sufficient to promote protection against chronic hypoxia-induced pulmonary hypertension. It may also indicate a potential contribution of the CRH-spleen axis to pulmonary hypertension, opening new lines of investigation and potential new targets for disease-modifying PH therapy.

## Data availability statement

The original contributions presented in the study are included in the article/supplementary material, further inquiries can be directed to the corresponding authors.

## Ethics statement

The animal study was reviewed and approved by University of Florida Institute Animal Care and Use Committee.

## Author contributions

AO designed and performed experiments, analyzed results, interpreted findings, and wrote the manuscript. MK, MA, and JH performed the experiments. AK and EK provided CRH ACE2KI mice, discussed and/or interpreted findings, and revised the manuscript. ER discussed findings and revised the manuscript. AB and MR supervised the study, discussed findings, and revised the manuscript. All authors contributed to the article and approved the submitted version.

## Funding

This research was supported by NIH grants K99HL165026, HL102033, R35HL150750, HL150750, HL142887, and HL142776 and AHA Postdoctoral Fellowship Grant number 20POST35210516.

## Conflict of interest

The authors declare that the research was conducted in the absence of any commercial or financial relationships that could be construed as a potential conflict of interest.

## Publisher’s note

All claims expressed in this article are solely those of the authors and do not necessarily represent those of their affiliated organizations, or those of the publisher, the editors and the reviewers. Any product that may be evaluated in this article, or claim that may be made by its manufacturer, is not guaranteed or endorsed by the publisher.
